# Knockdown of SOX18 inhibits the proliferation, migration and invasion of hepatocellular carcinoma cells

**DOI:** 10.3892/or.2015.4112

**Published:** 2015-07-07

**Authors:** GUIMING WANG, ZHIGANG WEI, HONGYAN JIA, WENBO ZHAO, GAOCHAO YANG, HAOLIANG ZHAO

**Affiliations:** 1 Department of Surgery, The First Hospital of Shanxi Medical University, Taiyuan 030001, P.R. China; 2 Department of Surgery, Shanxi Dayi Hospital, Taiyuan 030032, P.R. China

**Keywords:** SOX18, HCC, proliferation, invasion, chemokine, focal adhesion

## Abstract

Hepatocellular carcinoma (HCC) is one of the most common malignancies in the world. Recent studies have demonstrated that SOX18 is highly expressed in various types of cancer. In the present study, we found that SOX18 mRNA was overexpressed in HCC compared with non-tumorous tissues. We aimed to explore the effects of SOX18 siRNA on the proliferation, invasion and migration of two HCC cell lines, MHCC97H and HepG2, which overexpress SOX18. We found that SOX18 siRNA significantly inhibited the proliferation and induced cell cycle arrest at the G0/G1 phase. Results of the Transwell assay showed that the migration and invasion of the HCC cells were markedly impaired in the SOX18-knockdown cells. Gene set enrichment analysis (GSEA) showed that KEGG focal adhesion and chemokine signaling pathways were correlated with SOX18 expression. Furthermore, the mRNA and protein levels of RhoA, PDGFB, IGF1R, CCL2, CCL3 and CCL5 were decreased in the SOX18-knockdown cells. Importantly, we demonstrated that upregulation of SOX18 was associated with a poor outcome in HCC patients. These results indicate that SOX18 may serve as a prognostic factor and a promising therapeutic strategy for HCC.

## Introduction

Hepato growth factor associated with the metastasis cellular carcinoma (HCC) is the fifth most common malignancy in the world, with more than 748,000 new cases diagnosed annually. HCC is associated with a particularly poor prognosis. HCC ranks as the second cause of mortality from cancer and is associated with the shortest survival rate of any cancer (1,2). The overall poor survival rate of HCC patients is mainly ascribed to late disease diagnosis, which rules out curative surgery for the majority of patients (3). The prognosis for patients eligible for surgical resection remains dismal, due to the high potential for vascular invasion, metastasis and recurrence (4). The poor clinical outcome of HCC patients emphasizes the importance of a better understanding of the transcriptional activation of oncogenic signaling pathways and the control of cancer-associated genes.

The SOX family consists of a group of transcription factors that are defined by a highly conserved high-mobility group (HMG) DNA-binding domain (5,6). Knockout experiments demonstrated that SOX genes have a wide range of roles in development (7). Recently, a number of links have been found between SOX transcription factors and human related types of cancer (8). For instance, SOX7 (9–11) and SOX17 (12,13) act as tumor suppressors in various types of cancer through suppression of Wnt signaling. Contrastingly, SOX2 has been reported as a potential oncogene in breast and lung cancer (14,15). In the case of SOX18, overexpression of SOX18 has been found in gastric cancer, as compared to normal gastric tissues (16). Furthermore, SOX18 expression was found to be correlated with a poor clinical outcome of patients with ovarian (17), non-small cell lung cancer (18) or invasive ductal breast carcinoma (19). However, little is known concerning the expression pattern and biological functions of SOX18 in HCC.

In the present study, the SOX18 mRNA level was significantly increased in HCC tissues. Then, by siRNA-mediated silencing of SOX18 in HCC cell lines, we found that SOX18 is involved in the regulation of cell proliferation, cell cycle progression and metastasis. Furthermore, gene set enrichment analysis (GSEA) showed that KEGG focal adhesion and chemokine signaling pathways were correlated with SOX18 expression. Importantly, we demonstrated that upregulation of SOX18 is associated with a poor outcome in HCC patients. Collectively, the present study provides original documentation for the overexpression of SOX18 in HCC and it may be an effective therapeutic target for this disease.

## Materials and methods

### Patients and tissue samples

From 2006 to 2008, 75 patients admitted to Shanxi Dayi Hospital were enrolled in the present study. In each case, HCC and adjacent non-tumorous tissues were snap-frozen in liquid nitrogen and stored at −80°C until RNA extraction. Follow-up was completed in October 2013. Ethical approval for the present study was provided by the Independent Ethics Committee of Shanxi Dayi Hospital. Informed and written consent was obtained from all patients or their advisers according to the ethics committee guidelines.

### Cell lines

HepG2, BEL-7404, SMC-7721, MHCC-97L and MHCC-97H cells were obtained from the Cell Bank of Shanghai Biology Institute, Chinese Academy of Science (Shanghai, China). All culture media (Life Technologies, Carlsbad, CA, USA) were supplemented with 10% fetal bovine serum (FBS; Life Technologies), 100 mg/ml penicillin G and 50 *µ*g/ml streptomycin (Life Technologies). HepG2, BEL-7404, MHCC-97L and MHCC-97H cells were cultured in DMEM. SMC-7721 cells were cultured in RPMI-1640 medium.

### Silencing of SOX18 by small interfering RNA

The siRNA targeting position 1344–1362 (CUCUCUCAUACGCGUGUAU) of human SOX18 mRNA was synthesized. A non-specific scramble siRNA sequence was used as the negative control (NC). The siRNAs were transiently transfected into MHCC-97H and HepG2 cells using Lipofectamine 2000 (Invitrogen) according to the manufacturer's instructions. Assays were performed 48 h after transfection.

### Reverse transcription and real-time PCR

Total RNA was extracted using TRIzol reagent (Invitrogen) according to the manufacturer's instructions. cDNA was synthesized by using M-MuLV Reverse Transcriptase (Thermo Fisher Scientific, Rockford, IL, USA). Real-time PCR was performed using a standard SYBR-Green PCR kit (Thermo) protocol on an ABI 7300 real-time PCR machine (Applied Biosystems, Foster City, CA, USA). GAPDH served as an internal control. The gene expression was calculated using the ∆∆Ct method. All data represent the average of three replicates. The primers used are listed in Table I.

### Western blot analysis

Treated and untreated MHCC-97H and HepG2 cells were lysed in ice-cold radio immunoprecipitation assay buffer. Equal amounts of protein were separated via SDS-PAGE gel electrophoresis and electro-blotted onto a nitrocellulose membrane. Immunodetection of proteins was performed using specific antibodies. Densitometric analysis was performed with Image J software. Primary antibodies were obtained from the following companies: i) SOX18, PDGFB, IGF1R and CCL3 (Abcam, Cambridge, MA, USA); ii) CCL2, CCL5, RhoA and GAPDH, CST (Biotech, Danvers, MA, USA).

### Cell proliferation assay

Cell proliferation was measured using the Cell Counting Kit-8 (CCK-8) (Dojindo Lab, Kumamoto, Japan). Briefly, the treated and untreated MHCC-97H and HepG2 cells were seeded onto 96-well plates. At the indicated time-point, CCK8 solution was added to each well and incubated for 1 h. The optical density values (OD) were measured at 450 nm by using a microplate reader (Bio-Rad Laboratories Inc., Hercules, CA, USA). All experiments were performed in triplicates and repeated at least three times.

### Cell cycle distribution analysis

Propidium iodide (PI) staining was used to analyze DNA content. Treated and untreated MHCC-97H and HepG2 cells were harvested and labeled with PI by using previously described methods (20). DNA content was analyzed using a FACScan flow cytometry (BD Biosciences, San Jose, CA, USA) and the percentages of cells in the G0/G1, S and G2/M phases were determined with the FlowJo software (Tree Star). Experiments were performed in triplicate.

### Cell apoptosis analysis

The percentage of apototic cells was determined by double staining with Annexin V-fluorescein isothiocyanate (FITC) and PI. With or without siRNA transfection for 48 h, both adherent and floating cells were harvested, washed with PBS, pelleted and re-suspended in an incubation buffer containing Annexin V-FITC (BD Biosciences) and PI. The subsequent analysis was performed on a FACScan flow cytometry.

### Boyden chamber assay for migration and invasion

Quantitative cell migration and invasion assays were performed using 12-well Boyden chambers (Coring, NY, USA). For the migration assay, siRNA-transfected cells were serum-starved for 24 h, and then 5×10^4^ cells were seeded into the upper well of the Boyden chambers, with 500 ml of serum-free medium added to the lower chamber. After 24 h of incubation, the cells on the upper surface of the filter were completely removed. The remaining cells were washed with PBS, fixed in 4% paraformaldehyde and stained with 0.2% crystal violet. The migrated cells were observed under a Leica inverted microscope (Deerfield, IL, USA) and counted.

For the *in vitro* invasion assay, the upper well of the Boyden Chamber was pre-coated with 10 mg/ml Matrigel (BD Biosciences). The rest of the assay was performed as described above.

### Gene Set Enrichment Analysis (GSEA)

To gain further insight into the biological pathways involved in HCC pathogenesis through the SOX18 pathway, GESA, a method of analyzing and interpreting microarray and the data using biological knowledge (21), was performed using GSEA version 2.0 from the Broad Institute at MIT, as previously described (22,23). RNA-sequencing data of the HCC cohort were downloaded from The Cancer Genomic Atlas project (TCGA) and analyzed by GSEA. In the present study, GSEA firstly generated an ordered list of all genes according to their correlation with SOX18 expression and then a predefined gene set (signature of gene expression upon perturbation of certain cancer-related gene) receives an enrichment score (ES), which is a measure of statistical evidence rejecting the null hypothesis that its members are randomly distributed in the ordered list. The expression level of SOX18 was used as phenotype label and 'Metric for ranking genes' was set to Pearson's correlation. The KEGG gene set biological process database (c2.KEGG. v4.0) from the Molecular Signatures Database was used for enrichment analysis.

### Statistical analysis

Survival curves were obtained by the Kaplan-Meier method and the differences in survival between low and high SOX18 expression groups were analyzed with the log-rank test. The two-tailed Student's t-test was used to evaluate statistical differences between two groups. Statistical significance was set at P<0.05. Where appropriate, data are expressed as mean ± SD.

## Results

### Overexpression of SOX18 in HCC tissues is correlated with reduced survival

**Figure 1:**
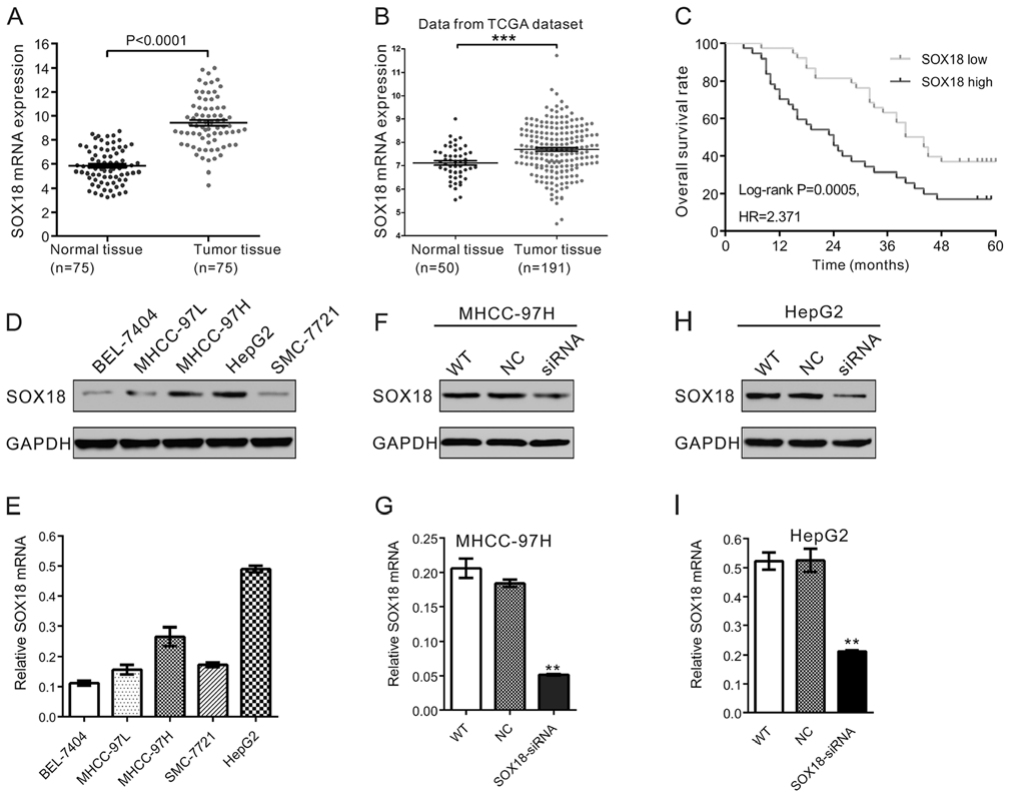
SOX18 is overexpressed in HCC tissues. (A) SOX18 mRNA level was significantly higher in HCC than that in non-tumorous tissues from patients admitted to Shanxi Dayi Hospital between 2006 and 2008 (P<0.0001). (B) SOX18 expression was significantly increased in the HCC tissues when compared with the adjacent tissues of patients from the TCGA dataset. (C) Kaplan-Meier survival analysis of HCC patients. The survival time of patients with a low SOX18 expression level was notably longer than that of patients with high SOX18 expression (P<0.01). (D and E) The SOX18 expression level in five HCC cell lines was analyzed by western blot analysis and real-time PCR. The silencing effect of SOX18-siRNA was evaluated by western blot analysis and real-time PCR in the MHCC-97H (F and G) and HepG2 (H and I) cells. WT, wild-type cells; NC, cells transfected with scrambled siRNA; siRNA, cells transfected with SOX18-siRNA. Data are based on at least 3 independent experiments and are shown as mean ± SD (^**^P<0.01 as compared with NC).

We first measured the SOX18 mRNA level in 75 patient HCC and adjacent non-tumorous tissues by real-time PCR. As shown in Fig. 1A, statistical analysis using the Student's t-test showed that SOX18 mRNA was significantly overexpressed in the HCC tissues when compared with that in the normal tissues (P<0.001).

In addition, we re-analyzed high throughput RNA-sequencing data of the HCC cohort of TCGA and also found a significant increase in SOX18 expression in the HCC tissues, and high SOX18 expression was compared with that in the normal tissues (Fig. 1B).

We next carried out Kaplan-Meier survival analysis to investigate the clinical outcome of HCC patients with low or high SOX18 expression. As shown in Fig. 1C, the survival time of the patients with high-SOX18-expressing tu mors was significantly shorter than that of patients with low-SOX18-expressing tumors (P<0.01). These results indicated that SOX18 expression was upregulated in the HCC tissues and was correlated with poor survival rate of these patients.

### Silencing of SOX 18 by RNA interference (RNAi)

To investigate the functions of SOX18 overexpression on HCC, we knocked down its expression in HCC cells by RNAi. We determined the protein and mRNA levels of SOX18 in five HCC cell lines, BEL-7404, MHCC-97H, MHCC-97L, HepG2 and SMC-7721, by western blot analysis and real-time PCR, respectively. Higher protein and mRNA levels of SOX18 were observed in two cell lines, MHCC-97H and HepG2 (Fig. 1D and E), which were selected for the RNAi experiment.

One siRNA targeting human SOX18 (SOX18-siRNA) and a negative control (NC, a non-specific scramble siRNA) were synthesized and used to transfect the MHCC-97H and HepG2 cells. The silencing effect of the siRNA was then evaluated by western blot analysis and real-time PCR. Our results indicated that SOX18-siRNA was able to efficiently suppress endogenous SOX18 expression in the MHCC-97H (Fig. 1F and G) and HepG2 cells (Fig. 1H and I).

### Silencing of SOX18 suppresses the proliferation, while induces G1-phase arrest and cell apoptosis in HCC cells

**Figure 2:**
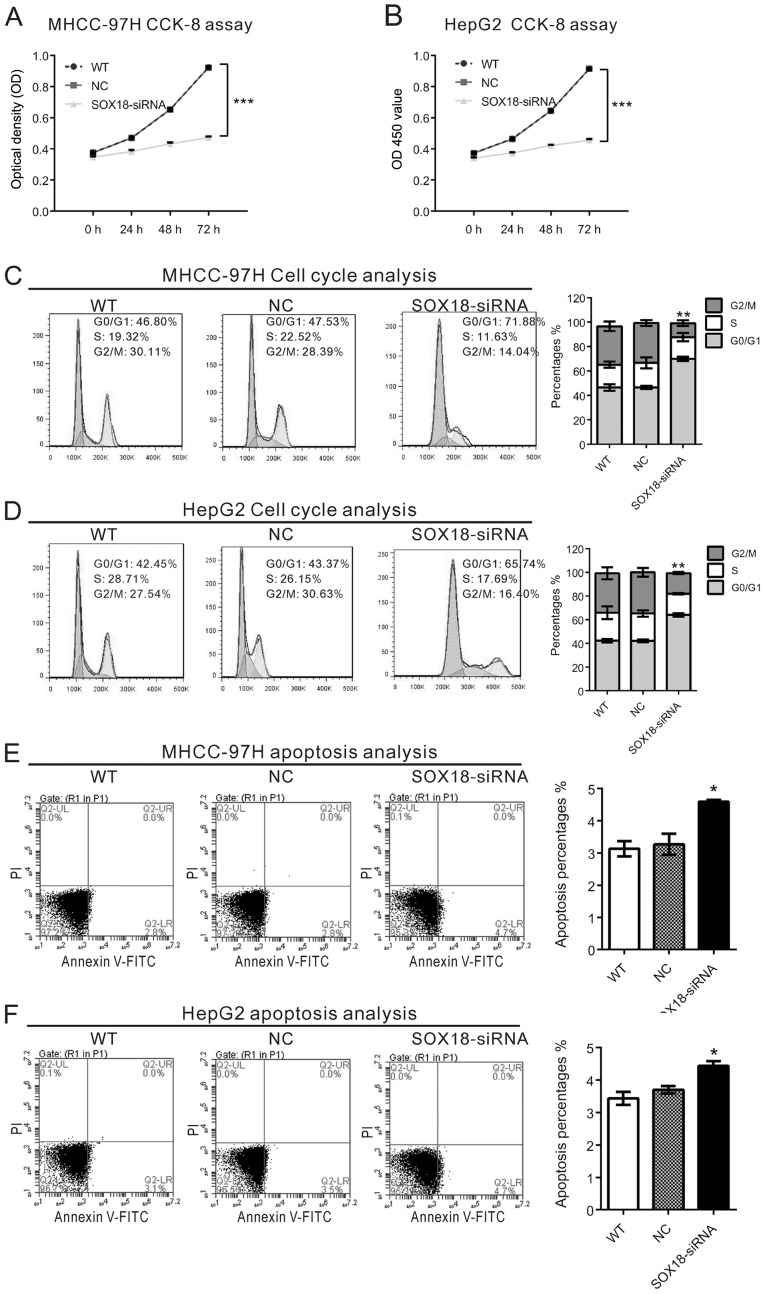
SOX18 RNAi suppresses the proliferation, while induces G1-phase arrest and cell apoptosis in the HCC cells. (A and B) Results of the CCK-8 assay performed in control cells and SOX18-knockdown cells. (C and D) Cell cycle profile. (E and F) Cell apoptosis analysis. WT, wild-type cells; NC, cells transfected with scrambled siRNA; siRNA, cells transfected with SOX18-siRNA. Data are at least three independent experiments and are shown as mean ± SD (^*^P<0.05, ^**^P<0.01 and ^***^P<0.001 as compared with NC).

To examine the effects of SOX18 silencing on the proliferation of HCC cells, the CCK-8 assay was performed. Cell proliferation of MHCC-97H (Fig. 2A) and HepG2 cells (Fig. 2B) transfected with SOX18-siRNA was notably impaired when compared to cell proliferation in the corresponding WT and NC cells. These results indicate that SOX18 may promote the proliferation of HCC cells.

The possible inhibitory effect of SOX18 knockdown on cell cycle progression was then evaluated. PI staining and flow cytometric analysis revealed that silencing of SOX18 in the MHCC-97H (Fig. 2C) and HepG2 cells (Fig. 2D) caused an increase in cells in the G1 phase and a corresponding decrease in cells in the S and G2/M phases. We also assessed the apoptotic function of SOX18 in the HCC cells by Annexin V-FITC/PI staining assay. As shown in Fig. 2E and F, MHCC-97H and HepG2 cells transfected with SOX18-siRNA exhibited slightly induced cell apoptosis compared with WT or NC cells. These results imply that the proliferation-promoting effect of SOX18 is mainly mediated by promoting cell cycle progression.

### Downregulation of SOX18 inhibits the motility and invasiveness of HCC cells

**Figure 3:**
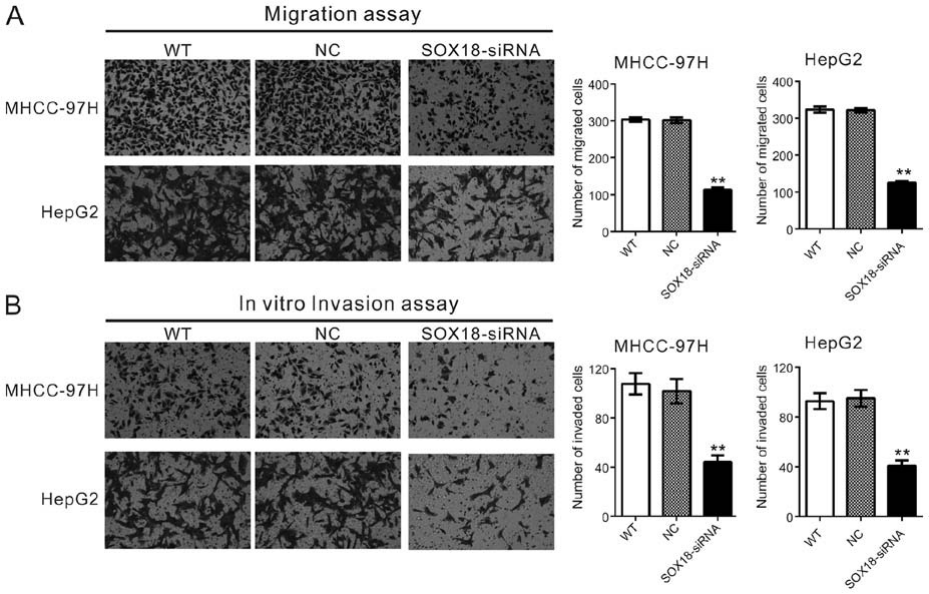
Silencing of SOX18 inhibits the migration and invasion of HCC cells. MHCC-97H and HepG2 cells were transfected with the indicated siRNA. (A) Migration assay in Transwell chambers. Cells that migrated from the upper well of a Transwell chamber into the lower well were stained, photographed and counted. Representative images are shown at the left. Quantitative results are shown at the right. (B) Invasion assay in Matrigel-coated Transwell chambers. Data are based on at least three independent experiments and are shown as mean ± SD (^**^P<0.01 as compared with NC).

To explore the involvement of SOX18 in cell motility, Transwell assays were carried out to quantitatively determine the effect of SOX18 on cell migration. As shown in Fig. 3A, similar numbers of WT and NC cells migrated to the lower face of the Transwell membrane (MHCC-97H: WT, 304±6; NC, 301±8; HepG2: WT, 323±8; NC, 322±6), whereas the SOX18-knockdown cells exhibited a strongly inhibited motility, with <40% cells migrating (MHCC-97H: 113±6; HepG2: 125±5).

We also investigated whether SOX18 affects the invasive ability of HCC cells by an *in vitro* invasion assay. As shown in Fig. 3B, depletion of SOX18 dramatically reduced the cell invasive ability when compared with that of the WT and NC cells. The number of invaded knockdown cells was ~43% of that of the control cells (MHCC-97H: WT, 108±9; NC, 102±10; SOX-siRNA, 44±5; HepG2: WT, 93±6; NC, 95±7; SOX-siRNA, 41±4). These data suggest that SOX18 promotes HCC cell invasion.

### Identification of genes and signaling-associated biological pathways and processes by GSEA

**Figure 4:**
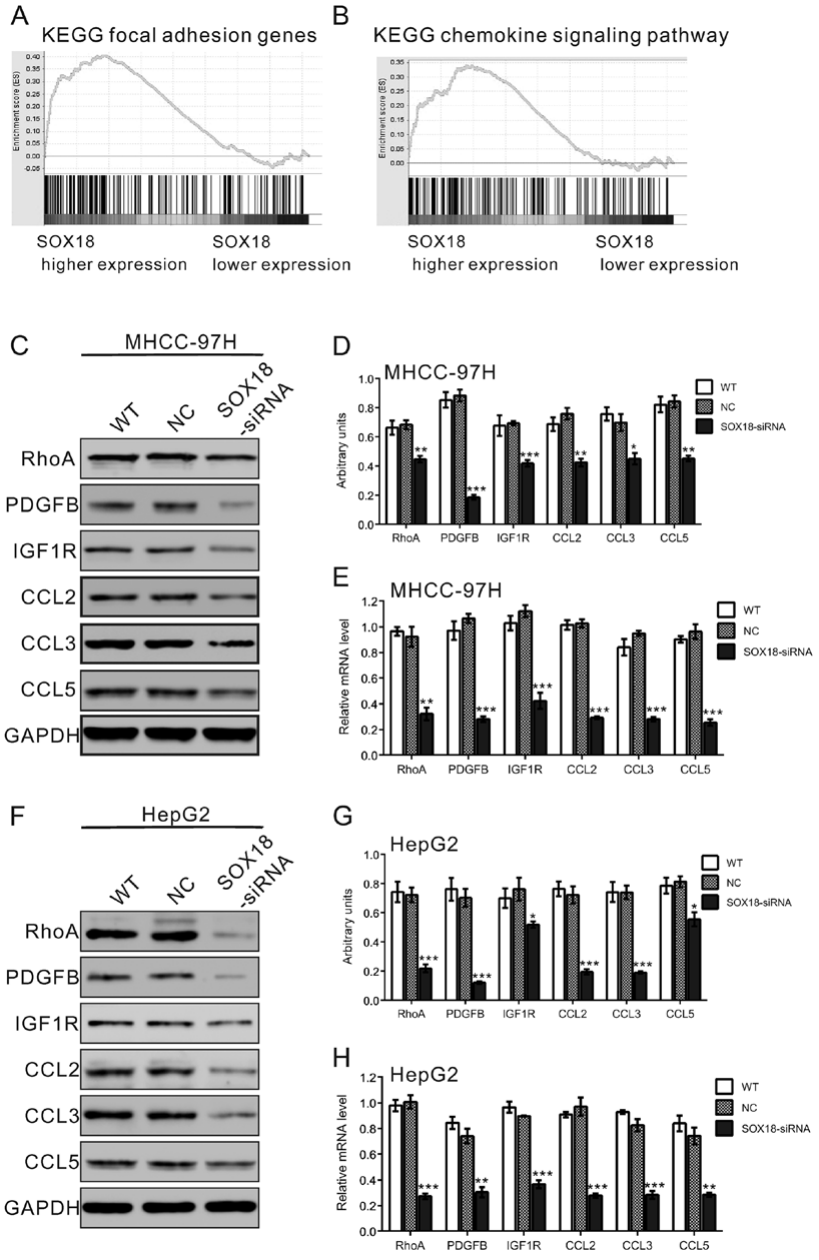
Effect of SOX18 on the expression of RhoA, PDGFB, IGF1R, CCL2, CCL3 and CCL5 in HCC cells. (A and B) GSEA identified KEGG focal adhesion and chemokine signaling pathways as regulatory targets of SOX18. After 48 h of SOX18 siRNA treatment, the protein levels of the indicated genes in the MHCC-97H (C and D) and HepG2 cells (F and G) were analyzed by western blot analysis. GAPDH was also detected as the control for sample loading. The mRNA levels of the indicated genes in the MHCC-97H (E) and HepG2 cells (H) were analyzed by real-time PCR. Data are based on at least three independent experiments and are shown as mean ± SD (^*^P<0.05, ^**^P<0.01 and ^***^P<0.001 as compared with NC).

To probe the SOX18-associated pathways on an unbiased basis, we performed GSEA using high throughput RNA-sequencing data of the HCC cohort from The Cancer Genome Atlas project (TCGA). GSEA is designed to detect coordinated differences in expression of predefined sets of functionally related genes. Among all the 188 predefined 'KEGG pathway' gene sets, the focal adhesion pathway and chemokine signaling pathways were identified as having a significant association with SOX18 expression in the HCC dataset (Fig. 4A and B).

### SOX18 siRNA regulates the mRNA and protein expression of RhoA, PDGFB, IGF1R, CCL2, CCL3 and CCL5 in HCC cells

The effects of SOX18 siRNA on the mRNA and protein levels of focal adhesion pathway genes (RhoA, PDGFB and IGF1R) and chemokine signaling pathway genes (CCL2, CCL3 and CCL5) were investigated. As shown in Fig. 4C–H, SOX18 siRNA treatment in the MHCC-97H and HepG2 cells significantly decreased the mRNA and protein levels of the detected genes in comparison with levels in the WT and NC groups.

## Discussion

The involvement of SOX genes in various cancers has been recently confirmed. Since most SOX genes behave as oncogenes in many human cancers, their targeting has great therapeutic potential. In the present study, we reported that the elevation of the SOX18 mRNA level was associated with the poor prognosis of patients with HCC. The *in vitro* experiments showed that knockdown of SOX18 expression inhibited the motile capacity of the HCC cells. These data indicated the diagnostic and therapeutic value of SOX18 for HCC.

Several studies (16–19) have reported the overexpression of SOX18 in gastric, ovarian, non-small cell lung and breast cancer. In the present study, SOX18 was identified as a potential biomarker for the diagnosis and prognosis of HCC. SOX18 mRNA was significantly upregulated in the HCC tissues when compared with adjacent non-tumorous tissues (Fig. 1A), which was confirmed by an independent HCC dataset from TCGA (Fig. 1B). More importantly, Kaplan-Meier survival analysis revealed that a high expression level of SOX18 was associated with a reduced patient survival rate (Fig. 1C).

Previous studies have suggested the promoting effect of SOX18 on cell proliferation of vascular smooth muscle cells (24) and MCF-7 breast cancer cells (25). In line with these findings, knockdown of SOX18 in the HCC cells significantly impaired cell growth (Fig. 2). Moreover, flow cytometric analysis showed that SOX18 knockdown induced G1 phase arrest and apoptosis of HCC cells (Fig. 2), which may have contributed to the inhibition of proliferation in the SOX18-knockdown cells. In addition, tumor metastasis was previously found to be inhibited in SOX18-deficient mice (26) and dominant-negative SOX18 shows an inhibitory effect on the migratory ability of MCF-7 cells (25). Consistent with these studies, we found that SOX18-siRNA treatment significantly decreased the migration and invasion capabilities of the HCC cells (Fig. 3), which suggest the role of SOX18 in promoting the metastasis of HCC.

In order to elucidate the possible mechanism involved in the SOX18-mediated inhibition of the motility of the HCC cells, we performed GSEA to identify the associated biological processes and signaling pathways using high throughput RNA-sequencing data of the HCC cohort of TCGA (Fig. 4). We found that the KEGG focal adhesion and chemokine signaling pathways were associated with SOX18 expression, which indicated that these two pathways play crucial role in the process of anti-proliferation, anti-migration and anti-invasion triggered by SOX18-siRNA.

The focal adhesion and chemokine signaling pathways (27) are involved in various cellular functions, including cell proliferation, motility, invasion and mortality. In the present study, the expression of KEGG focal adhesion genes (RhoA, IGF1R and PDGFB) and KEGG chemokine signaling genes (CCL2, CCL3 and CCL5) was evaluated in the SOX18-siRNA-treated HCC cells (Fig. 4). Small GTPase RhoA (28) and IGF1R (29) have long been recognized to play an important role in tumorigenesis and tumor progression. PDGFB is a growth factor associated with the metastasis of various types of human cancer (30). A growing body of research suggests the tumor-promoting roles of CCL2 (31,32), CCL3 (33) and CCL5 (32,34). Our data showed that SOX18 RNAi significantly downregulated the expression of detected genes, which indicating that SOX18 may execute its functions through regulating the expression of focal adhesion and chemokine signaling genes.

In conclusion, the present study proved for the first time that SOX18 plays a key role in the proliferation and metastasis of HCC cells. Moreover, SOX18 may regulate these biological processes through the focal adhesion and chemokine signaling pathways, thus providing useful information for the targeted therapy of HCC. As the SOX18 expression level is associated with patient survival, inhibition of SOX18 in tumor tissues may provide an effective therapeutic strategy.

## Figures and Tables

**Table I:** Primers sequences for the quantitative PCR.

Primer	Primer sequence	Size (bp)
SOX18	F: 5′-CGCGTGTATGTTTGGTTC-3′	
(NM_018419.2)	R: 5′-ATGTAACCCTGGCAACTC-3′	211
RhoA	F: 5′-GAGTGTTCAGCAAAGACCAAAG-3′	
(NM_001664.2)	R: 5′-TTGCAGCAAGGTTTCACAAG-3′	124
IGF1R	F: 5′-GAGCCTCCTGTGAAAGTG-3′	
(NM_000875.4)	R: 5′-GCATCCTGCCCATCATAC-3′	175
PDGFB	F: 5′-CTCGATCCGCTCCTTTGATG-3′	
(NM_002608.2)	R: 5′-AGGAAGTTGGCGTTGGTG-3′	249
CCL2	F: 5′-AACCGAGAGGCTGAGACTAAC-3′	
(NM_002982.3)	R: 5′-GGAATGAAGGTGGCTGCTATG-3′	125
CCL3	F: 5′-TTCCGTCACCTGCTCAGAATC-3′	
(NM_002983.2)	R: 5′-TGGCTGCTCGTCTCAAAGTAG-3′	188
CCL5	F: 5′-CCTCGCTGTCATCCTCATTG-3′	
(NM_002985.2)	R: 5′-ACTTGGCGGTTCTTTCGG-3′	195
GADPH	F: 5′-CACCCACTCCTCCACCTTTG-3′	
(NM_001256799.1)	R: 5′-CCACCACCCTGTTGCTGTAG-3′	110

## References

[1] Ferlay J, Shin HR, Bray F, Forman D, Mathers C, Parkin DM (2010). Estimates of worldwide burden of cancer in 2008: GLOBOCAN 2008. Int J Cancer.

[2] Jemal A, Bray F, Center MM, Ferlay J, Ward E, Forman D (2011). Global cancer statistics. CA Cancer J Clin.

[3] Pang RW, Poon RT (2007). From molecular biology to targeted therapies for hepatocellular carcinoma: The future is now. Oncology.

[4] Poon RTP, Fan ST, Wong J (2000). Risk factors, prevention, and management of postoperative recurrence after resection of hepatocellular carcinoma. Ann Surg.

[5] Wilson M, Koopman P (2002). Matching SOX: Partner proteins and co-factors of the SOX family of transcriptional regulators. Curr Opin Genet Dev.

[6] Bowles J, Schepers G, Koopman P (2000). Phylogeny of the SOX family of developmental transcription factors based on sequence and structural indicators. Dev Biol.

[7] Schepers GE, Teasdale RD, Koopman P (2002). Twenty pairs of sox: Extent, homology, and nomenclature of the mouse and human sox transcription factor gene families. Dev Cell.

[8] Dong C, Wilhelm D, Koopman P (2004). Sox genes and cancer. Cytogenet Genome Res.

[9] Li B, Ge Z, Song S, Zhang S, Yan H, Huang B, Zhang Y (2012). Decreased expression of SOX7 is correlated with poor prognosis in lung adenocarcinoma patients. Pathol Oncol Res.

[10] Stovall DB, Wan M, Miller LD, Cao P, Maglic D, Zhang Q, Stampfer MR, Liu W, Xu J, Sui G (2013). The regulation of SOX7 and its tumor suppressive role in breast cancer. Am J Pathol.

[11] Zhang Y, Huang S, Dong W, Li L, Feng Y, Pan L, Han Z, Wang X, Ren G, Su D (2009). SOX7, down-regulated in colorectal cancer, induces apoptosis and inhibits proliferation of colorectal cancer cells. Cancer Lett.

[12] Ye YW, Wu JH, Wang CM, Zhou Y, Du CY, Zheng BQ, Cao X, Zhou XY, Sun MH, Shi YQ (2011). Sox17 regulates proliferation and cell cycle during gastric cancer progression. Cancer Lett.

[13] Du YC, Oshima H, Oguma K, Kitamura T, Itadani H, Fujimura T, Piao YS, Yoshimoto T, Minamoto T, Kotani H (2009). Induction and down-regulation of Sox17 and its possible roles during the course of gastrointestinal tumorigenesis. Gastroenterology.

[14] Chen Y, Shi L, Zhang L, Li R, Liang J, Yu W, Sun L, Yang X, Wang Y, Zhang Y (2008). The molecular mechanism governing the oncogenic potential of SOX2 in breast cancer. J Biol Chem.

[15] Rudin CM, Durinck S, Stawiski EW, Poirier JT, Modrusan Z, Shames DS, Bergbower EA, Guan Y, Shin J, Guillory J (2012). Comprehensive genomic analysis identifies SOX2 as a frequently amplified gene in small-cell lung cancer. Nat Genet.

[16] Eom BW, Jo MJ, Kook MC, Ryu KW, Choi IJ, Nam BH, Kim YW, Lee JH (2012). The lymphangiogenic factor SOX 18: A key indicator to stage gastric tumor progression. Int J Cancer.

[17] Pula B, Kobierzycki C, Solinski D, Olbromski M, NowakMarkwitz E, Spaczynski M, Kedzia W, Zabel M, Dziegiel P (2014). SOX18 expression predicts response to platinum-based chemotherapy in ovarian cancer. Anticancer Res.

[18] Jethon A, Pula B, Olbromski M, Werynska B, Muszczynska-Bernhard B, Witkiewicz W, Dziegiel P, Podhorska-Okolow M (2015). Prognostic significance of SOX18 expression in non-small cell lung cancer. Int J Oncol.

[19] Pula B, Olbromski M, Wojnar A, Gomulkiewicz A, Witkiewicz W, Ugorski M, Dziegiel P, Podhorska-Okolow M (2013). Impact of SOX18 expression in cancer cells and vessels on the outcome of invasive ductal breast carcinoma. Cell Oncol (Dordr).

[20] Papavasiliou FN, Schatz DG (2000). Cell-cycle-regulated DNA double-strand breaks in somatic hypermutation of immunoglobulin genes. Nature.

[21] Subramanian A, Kuehn H, Gould J, Tamayo P, Mesirov JP (2007). GSEA-P: A desktop application for Gene Set Enrichment Analysis. Bioinformatics.

[22] Chen H, Xu J, Hong J, Tang R, Zhang X, Fang J-Y (2014). Long noncoding RNA profiles identify five distinct molecular subtypes of colorectal cancer with clinical relevance. Mol Oncol.

[23] Kapoor A, Yao W, Ying H, Hua S, Liewen A, Wang Q, Zhong Y, Wu CJ, Sadanandam A, Hu B (2014). Yap1 activation enables bypass of oncogenic Kras addiction in pancreatic cancer. Cell.

[24] Garcia-Ramirez M, Martínez-González J, Juan-Babot JO, Rodriguez C, Badimon L (2005). Transcription factor SOX18 is expressed in human coronary atherosclerotic lesions and regulates DNA synthesis and vascular cell growth. Arterioscler Thromb Vasc Biol.

[25] Young N, Hahn CN, Poh A, Dong C, Wilhelm D, Olsson J, Muscat GE, Parsons P, Gamble JR, Koopman P (2006). Effect of disrupted SOX18 transcription factor function on tumor growth, vascularization, and endothelial development. J Natl Cancer Inst.

[26] Duong T, Proulx ST, Luciani P, Leroux JC, Detmar M, Koopman P, Francois M (2012). Genetic ablation of SOX18 function suppresses tumor lymphangiogenesis and metastasis of melanoma in mice. Cancer Res.

[27] Locati M, Deuschle U, Massardi ML, Martinez FO, Sironi M, Sozzani S, Bartfai T, Mantovani A (2002). Analysis of the gene expression profile activated by the CC chemokine ligand 5/RANTES and by lipopolysaccharide in human monocytes. J Immunol.

[28] Struckhoff AP, Rana MK, Worthylake RA (2011). RhoA can lead the way in tumor cell invasion and metastasis. Front Biosci.

[29] Adams TE, McKern NM, Ward CW (2004). Signalling by the type 1 insulin-like growth factor receptor: Interplay with the epidermal growth factor receptor. Growth Factors.

[30] Jechlinger M, Sommer A, Moriggl R, Seither P, Kraut N, Capodiecci P, Donovan M, Cordon-Cardo C, Beug H, Grünert S (2006). Autocrine PDGFR signaling promotes mammary cancer metastasis. J Clin Invest.

[31] Zhang J, Patel L, Pienta KJ (2010). CC chemokine ligand 2 (CCL2) promotes prostate cancer tumorigenesis and metastasis. Cytokine Growth Factor Rev.

[32] Soria G, Ben-Baruch A (2008). The inflammatory chemokines CCL2 and CCL5 in breast cancer. Cancer Lett.

[33] Yang X, Lu P, Fujii C, Nakamoto Y, Gao JL, Kaneko S, Murphy PM, Mukaida N (2006). Essential contribution of a chemokine, CCL3, and its receptor, CCR1, to hepatocellular carcinoma progression. Int J Cancer.

[34] Luboshits G, Shina S, Kaplan O, Engelberg S, Nass D, Lifshitz-Mercer B, Chaitchik S, Keydar I, Ben-Baruch A (1999). Elevated expression of the CC chemokine regulated on activation, normal T cell expressed and secreted (RANTES) in advanced breast carcinoma. Cancer Res.

